# The Principles, Etc. of Modern Surgery

**Published:** 1860-11

**Authors:** 


Art. VII.—*The Principles and Practice of Modern Surgery.* By Robert Druitt, Licentiate of the Royal College of Physicians, London, etc. etc. 8vo., pp. 695. With Four Hundred and Thirty-two Illustrations. Blanchard & Lea: Philadelphia, 1860.

Few works on any branch of medical science have met with such signal success as the Principles and Practice of Surgery, by Mr. Robert Druitt. Originally written when the author was quite a young man, hardly out of the lecture-room, each successive edition has been carefully revised and enlarged until now, when it has reached its eighth issue, it forms a portly and closely-printed octavo of nearly seven hundred pages, illustrated by upwards of four hundred wood-cuts. The publishers have spared no pains to bring out the present edition in a superior manner, and the work, we have no doubt, will continue to be sought after by American students as a reliable guide during their attendance upon lectures. The great popularity of the work at once attests its excellence. It has, unquestionably, some faults, but these are, in the main, of a minor character. The author, for example, has not, in our judgment, treated some of his topics with that minuteness of detail so essential in an elementary book; nor has he done sufficient justice to American surgeons, since, notwithstanding their great achievements in the operative part of the subject, he rarely refers to any of them. This is the more remarkable when we consider the pains which have been taken in this country to disseminate Mr. Druitt's work.

The present edition is accompanied by notes by a young physician of Philadelphia, who has, in some degree, atoned for the omissions here spoken of, but hardly to the extent that could have been desired. He has very modestly withheld his name from the title-page, a circumstance for which he deserves our cordial acknowledgments, for we sincerely hope that we shall see no more of these "pedicular excrescences," to use the language of Dr. Oliver Wendell Holmes, in connection with foreign reprints.

